# Behavior of Spoilage Bacterial Communities in Different Cuts of Enshi Black Pork under Refrigerated Storage (4 °C)

**DOI:** 10.3390/foods13132081

**Published:** 2024-07-01

**Authors:** Ying Zhang, Jiang Yang, Lijuan Peng, E Liao, Haibin Wang

**Affiliations:** 1 College of Food Science and Engineering, Wuhan Polytechnic University, Wuhan 430023, China; zy920506@sina.com (Y.Z.); yj19972024@163.com (J.Y.); lijuan_peng@hotmail.com (L.P.); leoneason@126.com (E.L.); 2 Key Laboratory for Processing and Transformation of Agricultural Products, Wuhan Polytechnic University, Wuhan 430023, China

**Keywords:** Enshi black pork, pork cut, bacterial community, spoilage bacteria, refrigerated storage

## Abstract

The Enshi black pig is a Chinese native breed known for its rich nutrition content and high quality, which has notable traction in the consumer market. In this study, the potential impact of the main commercial cuts from Enshi black pork carcasses (ham, loin, and belly) on the bacteria community of spoiled meat under refrigerated storage (4 °C) was assessed by using a high-throughput sequencing method. Moreover, the spoilage potential of isolated strains from spoiled pork was investigated. The results demonstrated significant differences (*p* < 0.05) in bacterial community diversity and composition among spoiled ham, loin, and belly samples. Linear discriminant analysis effect size (LEfSe) analysis revealed a total of 20 significantly different potential bacterial biomarkers, with the dominant genera of *Pseudomonas*, *Psychrobacter*, *Shewanella* and *Carnobacterium*. Additionally, *C. divergens* THT1-5, isolated from spoiled ham samples, displayed cold adaptation and higher spoilage potential in Enshi black pork. These findings are helpful for identifying key factors contributing to spoilage in Enshi black pork and developing strategies to inhibit bacterial growth during preservation.

## 1. Introduction

Pork, being one of the most common meat products worldwide, has a significant influence across various regions [1,2,3]. In particular, China stands out as a major player in pork production and consumption, boasting a substantial market [4,5]. Recent data from 2020 showed that the global pork output has reached 97.88 million tons, with China accounting for an impressive 38.83% share, surpassing output levels in other countries by a significant margin [6]. Within China, the Enshi black pig breed has gained notable traction in the consumer market due to its unique local characteristics [7,8]. This breed is known for its high-quality meat, rich in nutrition and high in intramuscular fat content, characterized by high levels of unsaturated fatty acids, premium fat quality, a bright red color, and tender meat texture [9]. In addition to breed, factors such as age, fatness and leanness as well as anatomical location play crucial roles in shaping the quality of pork cuts [10]. Notably, pork ham, loin, and belly emerge as important commercial cuts derived from pork carcasses, showcasing variations in nutritional content, culinary properties, taste, and flavor.

During storage, microbial spoilage is commonly considered the primary factor contributing to the deterioration of meat quality [11,12]. The rich protein and fat content in pork make it easy for microorganisms to thrive, thereby affecting the overall quality of meat [13,14]. The spoilage bacteria in meat are conventionally categorized as Gram-negative rods, Gram-positive spore formers, lactic acid bacteria, and other Gram-positive bacteria [12]. The core spoilage microbiota consists of *Acinetobacter* spp., *Staphylococcus* spp., *Streptococcus* spp., as well as Proteobacteria, notably *Pseudomonas* spp., and members of Enterobacteriaceae [15]. Furthermore, different pork cuts possess varying ratios of protein to fat, along with distinct cutting methods and processing environments, which are expected to exert a significant influence on meat spoilage during storage. Therefore, it is imperative to investigate the microbial profile of different pork cuts, particularly focusing on spoilage bacteria throughout refrigerated storage. The expanding field of high-throughput DNA sequencing offers fresh perspectives on the overall dynamics of bacterial communities in pork spoilage [16].

This study aims to assess the potential impact of the main commercial cuts from Enshi black pork carcasses (ham, loin, and belly) on the bacterial community of spoiled meat under refrigerated storage (4 °C) using high-throughput sequencing. Additionally, the spoilage potential of isolated strains from spoiled pork will be investigated. An important and innovative perspective will be used to describe the relationship between bacterial community diversity and different pork cuts. The findings of this study will help identify key factors contributing to spoilage in Enshi black pork and develop strategies to inhibit bacteria growth during preservation.

## 2. Materials and Methods

### 2.1. Samples Preparation

The Enshi black pork cuts (ham, loin, and belly) were provided by the Hubei Sile Animal Husbandry Group Company (Enshi, China). The pigs were slaughtered in a commercial abattoir by standard procedures, and pork cuts were collected from 30 barrow carcasses. They were vacuum-packed and then transported to the laboratory under refrigerated conditions at 4 °C. Upon arrival, the pork ham, loin, and belly were promptly segmented into 20 mm-thick pieces, eliminating tendons, membranes, connective tissues, and other non-essential components. Subsequently, the pork cuts were meticulously packed into 250 g trays and sealed with plastic wrap. These packaged pork cuts of 3 groups (ham, loin, and belly groups) were stored in a 4 °C refrigerator until spoilage, and samples were collected on days 0, 2 and 4. This study was conducted with three batches (10 carcass × 3 times).

### 2.2. Total Viable Count (TVC) Analysis

Following the China National Food Safety Standard (GB 4789.2-2022) [17], the total viable count (TVC) was assessed. A sterile homogeneous bag was filled with 225 mL of sterile saline (0.85%), and 25 g of pork was cut and placed inside. The bag was then put into in a homogenizer and mixed for 5 min. TVCs were determined using plate count agar (PCA) (Aoboxing Biotech Company, Beijing, China) at 37 °C for 48 h. The results were presented as Log CFU/g. Each sample was measured in triplicate.

### 2.3. Total Volatile Basic Nitrogen (TVB-N) Content Analysis

The total volatile basic nitrogen (TVB-N) content in pork cuts was determined according to the semi-micro Kjeldahl method outlined in the China National Food Safety Standard (GB 5009.228-2016) [18]. A measure of 10 g of chopped pork was soaked in 100 mL of distilled water for 30 min and then filtered into a distillation tube. Subsequently, 1 g of magnesium oxide was added to the distillation tube, which was immediately connected to the automatic Kjeldahl nitrogen determinator (Hanon Instruments Company, Jinan, China) for analysis. The distillate was collected in a flask containing 10 mL of 20 g/L boric acid and titrated with 0.01 mol/L of HCl to determine the TVB-N content accurately. The results are expressed as mg/100 g. Each sample was measured in triplicate.

### 2.4. Bacterial DNA Extraction, PCR Amplification and Sequencing

The bacterial DNA was extracted from pork samples (3 samples × 9 replicates) using the commercial Microbiome DNA Isolation Kit (QIAGEN, Hilden, Germany). The extracted DNA was subjected to quality and purity inspection through 2% agarose gel electrophoresis and UV spectrophotometry (Nano Drop ND-2000C, Thermo Fisher Scientific, Waltham, MA, USA). DNA samples showing clear bands and an OD_260/280_ ratio reading between 1.8 and 2.0 were chosen for further experiments. 

Following the DNA extraction process, the V3–V4 variable region of 16S rDNA from the samples underwent amplification using universal primers 338F (5′-ACTCCTACGGGAGGCAGCAG-3′) and 806R (5′-GGACTACHVGGGTWTCTAAT-3′), following the method described by Li et al. [19] for PCR amplification. The PCR products were purified utilizing the QIAquick Gel Extraction Kit (QIAGEN, Germany) and quantified with a Qubit^@^ 2.0 Fluorometer (Thermo Fisher Scientific, Waltham, MA, USA). The constructed paired-end (PE) library (300 bp) was sequenced on the Illumina MiSeq platform by Majorbio Bio-Pharm Technology Company (Shanghai, China).

### 2.5. Bioinformatics Analysis

The raw reads were filtered by the QIIME platform (v1.8.0, http://qiime.sourceforge.net/ (accessed on 1 March 2024)) [20], followed by alignment and calibration of sequences using pyNAST (t http://github.com/qiime/pynast/) (accessed on 1 March 2024)) [21]. The processed sequences were subjected to cluster classification with 97% similarity, leading to the completion of operational taxonomic units (OTUs). Utilizing the Chimera Slayer algorithm, OTUs containing chimeric sequences were removed [22,23]. The representative sequences were selected from each OTU and compared with RDP (Ribosomal Database Project, v2.2, http://rdp.cme.msu.edu/ (accessed on 1 March 2024)), Greengenes (v 13.5, http://greengenes.secondgenome.com/ (accessed on 1 March 2024)) and Silva (v138, http://www.arb-silva.de/ (accessed on 1 March 2024)) databases to assign their taxonomic classification [24]. Based on the phylogenetic tree constructed using FastTree 2 software (http://www.microbesonline.org/fasttree (accessed on 1 March 2024)) [25], α-diversity analysis was conducted, encompassing calculations of Chao1, observed species, Shannon, and Simpson indices. Chao1 and observed species indices assess bacterial community richness, while the Shannon and Simpson indices evaluate bacterial community diversity. These indices are crucial for evaluating bacterial community diversity and abundance. β-diversity analysis was performed at the genus level in samples performed using weighted and unweighted UniFrac distance [26]. Linear discriminant analysis effect size (LEfSe) analysis was carried out using LEfSe software (v 1.0, http://huttenhower.sph.harvard.edu/galaxy/root?tool_id=lefse_upload) (accessed on 1 March 2024) [27].

### 2.6. Spoilage Bacteria Isolation and Identification

The isolation of bacteria from spoiled samples of different pork cuts was performed using the following procedure: 0.1 mL of the chopped pork suspension was evenly spread onto PCA, after which the plates were incubated at 30 °C for 48 h. Subsequently, single colonies exhibiting distinct morphological characteristics, sizes and colors were selected and transferred to fresh PCA plates for purification. The purified cultures were preserved as glycerol suspensions (20%, *v*/*v*) and stored at −80 °C.

The chromosomal DNA of purified strains was extracted using a DNA extraction kit (Sangon Biotech, Shanghai, China). Subsequently, the PCR amplification of the 16S rRNA gene was conducted following the protocol outlined by Ma et al. [28]. The purified PCR products were sequenced using an ABI PRISM 3730XL automatic sequencer (Thermo Fisher Scientific, Waltham, MA, USA). To determine an approximate phylogenetic affiliation of each strain, the obtained sequences were compared with existing 16S rRNA gene sequences from GenBank through the BLAST nucleotide search tool on NCBI (http://blast.ncbi.nlm.nih.gov/ (accessed on 1 March 2024)).

### 2.7. Spoilage Potential Evaluation of Isolated Strains

Fresh Enshi black pork cuts (ham, loin, and belly) weighing 250 g were first placed on a clean, sterile bench and wiped with 75% alcohol disinfect. The pork cuts were drained and then immersed in the bacterial solution of isolated strains (10^6^ CFU/mL) for 30 s. Subsequently, they were drained again and carefully transferred into sterile bags, which were then stored under refrigeration conditions at 4 °C. The strains *Carnobacterium divergens* NHT2-5 and THT1-5 were inoculated separately with sterile pork ham. In the sterile pork loin, the strains *Pseudomonas taetrolens* NLJ3 and *C. divergens* TLJ2-6 were individually inoculated. Additionally, the strain *P. protegens* NWH1-3 was inoculated with sterile pork belly. Throughout the storage of pork, the TVC and TVB-N content were measured daily to evaluate the spoilage potential of the different strains.

### 2.8. Statistical Analysis

The Mann–Whitney test and Tukey’s multiple comparisons test were used to assess statistically significant differences with a significance level of *p* < 0.05, employing IBM SPSS 20 software (IBM Corporation, New York, NY, USA). Data analyses and graphics of the bacterial community were conducted using R software (v 4.2.0, https://www.r-project.org/ (accessed on 1 March 2024)).

## 3. Results and Discussion

### 3.1. Spoiled Black Pork Quality

**Figure 1:**
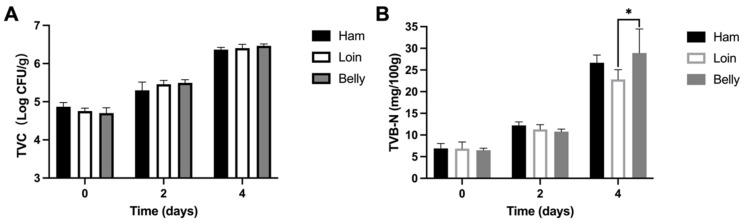
Evolution of total viable bacteria counts (TVCs) (**A**) and total volatile basic nitrogen (TVB-N) contents (**B**) in different cuts of black pork (ham, loin, and belly) during storage at 4 °C. Statistical significance is denoted by a star (*, *p* < 0.05).

The TVC and TVB-N content in various cuts of black pork (ham, loin, and belly) during storage at 4 °C are illustrated in Figure 1. The TVC in all samples rose significantly from 10^4^ CFU/g in fresh pork to 10^6^ CFU/g on the fourth day (*p* < 0.05) (Figure 1A). The initial TVB-N content of fresh pork was approximately 7 mg/100 g and over 20 mg/100 g in all samples on the fourth day of storage (Figure 1B). The increasing TVB-N content indicated the degradation of proteins in meat and enzymatic decarboxylation of specific amino acids by spoilage bacteria [29]. All samples exhibited correlated increasing trends in both TVC and TVB-N content, which aligns with previous findings [30]. As per the China National Food Safety Standard (GB/T 9959.2-2008) [31], the upper tolerable limits for the TVC and TVB-N content in fresh pork stand at 10^6^ CFU/g and 15 mg/100 g, respectively. The data indicated that the TVC and TVB-N content in the black pork stored on trays surpassed the threshold for spoilage under refrigeration storage (4 °C) by the fourth day. Furthermore, the TVB-N content in loin samples on the fourth day was significantly lower than that in ham and belly samples (*p* < 0.05). No noteworthy distinction was observed in TVC among the different cuts of black pork during storage (*p* > 0.05). These results suggested variations in spoilage among pork ham, loin, and belly, which may be attributed to different ratios of protein to fat.

### 3.2. Sequencing Results and Diversity Indices of Black Pork

**Figure 2:**
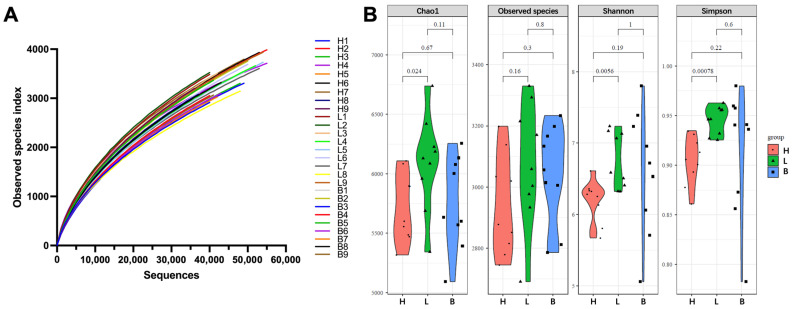
Rarefaction curves based on observed species (**A**) and alpha-diversity index (**B**) in spoiled black pork. H, ham; L, loin; B, belly. Nine replicates were represented by the numbers 1–9. The numbers above the lines indicate the statistical significance (*p*).

In this study, a total of 1,319,430 sequences were obtained from 27 samples of black pork, yielding an average of 48,867 sequences per sample. After removing chimeras, 54,934 OTUs were classified as 6 phyla, 13 classes, 25 orders, 42 families and 68 genera. As illustrated in Figure 2A, with the increase in the sequencing amount, the rarefaction curve based on observed species showed a gradual decrease in slope, as previously reported [28]. This decline suggested that species diversity experienced minimal changes as the sequencing amount increases, indicating that the sequencing volume was sufficient to analyze the bacterial community structure of the samples. Figure 2B presented the α-diversity of the bacterial community in all samples, encompassing Chao1, observed species, Shannon, and Simpson indices. The results revealed significant differences in Chao1, Shannon and Simpson indices between ham and loin samples (*p* < 0.05), indicating that the diversity and richness of the bacterial community in the loin were significantly higher than those in the ham. Conversely, no significant difference was observed between the ham and belly samples across the four indices (*p* > 0.05). Similarly, the comparison between the loin and belly samples also indicated no significant variation (*p* > 0.05).

### 3.3. Bacterial Community Composition of Black Pork

**Figure 3:**
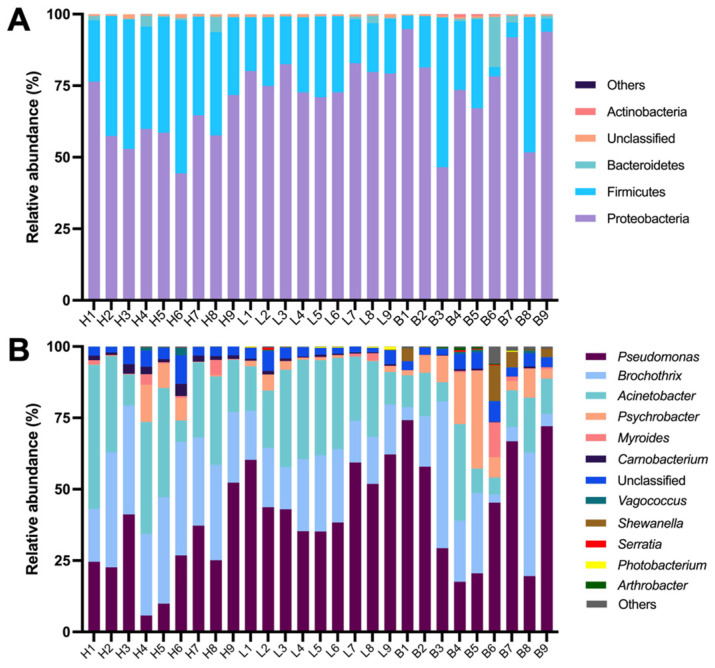
Relative abundance of bacterial community based on 16S rDNA sequencing on the phylum (**A**) and genus (**B**) level in spoiled black pork. H, ham; L, loin; B, belly. Nine replicates were represented by the numbers 1–9.

The dominant bacterial phyla in spoiled black pork were Proteobacteria, Firmicutes and Bacteroidetes, as illustrated in Figure 3A. These phyla collectively accounted for 99% of the bacterial community, a finding consistent with previous reports [32]. Proteobacteria, a Gram-negative staining bacterium commonly found in food, emerged as the most abundant taxa in the samples. Moreover, the relative abundance of Proteobacteria in ham samples (60.41 ± 9.04%) was significantly higher compared to that in loin and belly samples (*p* < 0.05). Conversely, the relative abundance of Firmicutes in samples of ham (37.34 ± 8.97%) was significantly lower than that in loin and belly samples (*p* < 0.05). These results illustrated differences in bacterial community composition at the phylum level between ham, loin, and belly.

A total of 68 genera were detected from all the samples at the genus level (Figure 3B). The dominant genera in the samples were *Pseudomonas*, *Brochothrix*, *Acinetobacter*, *Psychrobacter*, *Myroides* and *Carnobacterium*, as previously reported [33]. *Pseudomonas*, belonging to Proteobacteria, is an obligate aerobic Gram-negative bacterium that can thrive in a temperature range of 2 °C to 35 °C and is widely distributed in food [34,35]. This genus is famous for its protein-hydrolyzing ability in meat and for producing ethyl caproate, ethyl octanoate, and 1-octene-3-ol, which greatly impact the flavor of meat [34,36]. The relative abundance of *Pseudomonas* in ham samples (27.29 ± 13.81%) was significantly lower than that in loin and belly samples (*p* < 0.05). *Brochothrix*, a psychrophilic Gram-positive facultative anaerobe from Firmicutes phylum, is commonly found in fresh meat, particularly in meat products stored at low temperature [37]. This bacterium has been shown to produce volatile aldehydes, ketones, esters, and alcohols in meat by utilizing substances like glucose and glycerol, leading to meat discoloration and undesirable flavors [38]. The relative abundance of *Brochothrix* in ham samples (32.38 ± 7.01%) was significantly higher than that in loin and belly samples (*p* < 0.05). *Acinetobacter*, a Gram-negative coccus with a strict oxygen requirement, is a common spoilage bacterium present in fish and poultry meat [39]. Research indicated that *Acinetobacter* has limited putrescence capability in food but can enhance the putrescence ability of *Pseudomonas* and *Shewanella* [40]. Moreover, while the relative abundance of *Shewanella* was low in ham and loin samples (both less than 0.01%), it was notably higher in belly samples (up to 2.84%). This could be attributed to the ability of *Shewanella* to produce lipase, enabling it to metabolize the abundant fat in belly cuts [41]. To explore the diversity of the bacterial community across all samples, a heatmap illustrating the bacterial community structure and composition of the top genera was included in Figure S1, highlighting variations in the top genera among different cuts of black pork.

Analysis of sequencing data in Figure S2 demonstrated the presence of over 2000 OTUs in each sample. Among these, a total of 227 core OTUs were consistently detected across all samples. Notably, 10 core OTUs exhibited a relative abundance greater than 1.00%, including OTU32424 (19.77%), OTU22093 (7.46%), OTU48323 (5.49%), OTU47539 (5.18%), OTU16617 (4.12%), OTU31622 (3.69%), OTU6834 (3.27%), OTU11126 (3.23%), OTU6774 (2.72%) and OTU35263 (1.26%). The taxonomic classification revealed that these 10 core OTUs belonged to 4 distinct genera. Specifically, OTU32424 was identified as *Brochothrix*, while OTU22093, OTU48323, OTU35263, OTU6774 and OTU16617 were categorized as *Pseudomonas*. Additionally, OTU47539, OTU31622 and OTU6834 were classified under *Acinetobacter*, with OTU11126 assigned to *Psychrobacter*. These results suggested that *Pseudomonas*, *Brochothrix*, *Acinetobacter* and *Psychrobacter* comprised the predominant bacterial genera in spoiled black pork stored at 4 °C, consistent with findings in minced pork [42] and pork [43]. It is plausible that these bacteria utilize nutrients in raw meat to undergo metabolic processes, potentially leading to the production of various compounds such as aldehydes, ketones, alcohols, and ethers [44]. These metabolic activities could consequently influence the flavor and sensory quality of fresh meat.

### 3.4. Comparative Analysis of Bacterial Community

**Figure 4:**
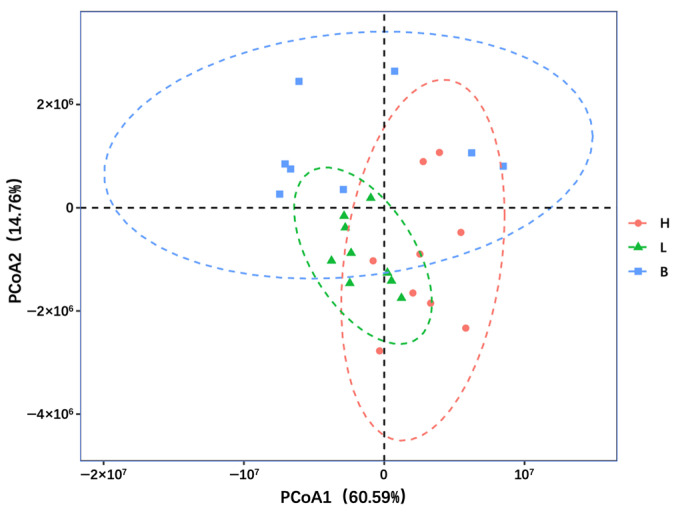
Principal coordinate analysis (PCoA) of bacterial community at the genus level in spoiled black pork based on unweighted UniFrac distance. H, ham; L, loin; B, belly.

The β-diversity analysis of 27 samples (3 groups × 9 replicates) of spoiled black pork at the genus level, shown in Figure 4, was clustered through principal coordinate analysis (PCoA) based on unweighted UniFrac distance. The first principal coordinate component (PCoA1) accounted for 60.59% of the variability, while the second principal coordinate component (PCoA2) explained 14.76% of the variability. Moreover, disparities were observed among the ham, loin, and belly samples, with samples within the same group tending to cluster together. The Mann–Whitney test results indicated significant differences (*p* < 0.05) in distances between the three groups of samples, suggesting that the bacterial communities varied across different cuts of black pork. These differences may be attributed to varying nutrient compositions and levels of spoilage in the different pork cuts [45].

**Figure 5:**
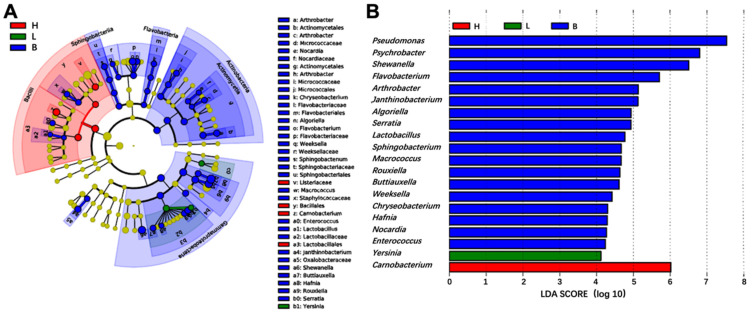
Linear discriminant analysis effect size (LEfSe) showing microbiome differences among three groups (H, L and B) of samples at the genus levels. (**A**) LEfSe cluster diagram. (**B**) LEfSe linear discriminant analysis (LDA) diagram. Bacterial taxa were significantly discriminant on the genus level with LDA scores larger than 4.0. H, ham; L, loin; B, belly.

LEfSe analysis was performed to identify potential biomarkers at the genus levels among the three groups of black pork samples (Figure 5). The cladogram of LEfSe analysis (Figure 5A) and linear discriminant analysis (LDA) results (Figure 5B) revealed a total of 20 bacterial genera with significantly different abundances detected from the three groups of samples (*p* < 0.05). In the belly samples, 18 genera showed notable differences, including *Pseudomonas*, *Psychrobacter*, *Shewanella*, *Flavobacterium*, *Arthrobacter*, *Janthinobacterium*, *Serratia*, *Algoriella*, *Lactobacillus*, *Sphingobacterium*, *Rouxiella*, *Macrococcus*, *Buttiauxella*, *Chryseobacterium*, *Enterococcus*, *Weeksella*, *Nocardia*, and *Hafnia*. One potential biomarker, *Carnobacterium*, was observed in the ham samples, while *Yersinia* was the potential biomarker in the loin samples. *Carnobacterium*, a member of lactic acid bacteria, can grow under diverse storage conditions and is commonly found in various fish and meats. Although some strains may contribute to meat and seafood spoilage, their prevalence is currently low [38,46]. On the other hand, *Yersinia*, known as a cryophilic bacterium, has been frequently isolated from pork lymph nodes, intestines, and feces. This pathogen can withstand and propagate in cold temperatures, making it a potential foodborne hazard that poses an infection risk for consumers [46].

### 3.5. Isolation and Identification of Strains in Spoiled Black Pork

In Table 1, the strains isolated in spoiled black pork from different cuts (ham, loin, and belly) were identified. A total of 50 strains were isolated from spoiled samples of ham, loin, and belly, representing 14 genera, with the majority belonging to *Carnobacterium* (11 strains), *Serratia* (10 strains), *Hafnia* (5 strains) and *Aeromonas* (5 strains). Combined with the high-throughput sequencing analysis, the dominant strains from each sample were chosen for spoilage potential assessment. For ham samples, the strains *C. divergens* NHT2-5 and THT1-5 from *Carnobacterium* genus were selected for evaluation. In the loin samples, *Yersinia*, a potential biomarker, was not isolated, possibly due to its low abundance [47]. Consequently, the strains *P. taetrolens* NLJ3 and *C. divergens* TLJ2-6, both with dominant genera, were selected for spoilage potential evaluation from the loin samples. Furthermore, in the belly samples, the strain *P. protegens* NWH1-3, a member of the top potential biomarker, *Pseudomonas*, was selected for the subsequent assessment.

### 3.6. Spoilage Potential of Isolated Strains

**Figure 6:**
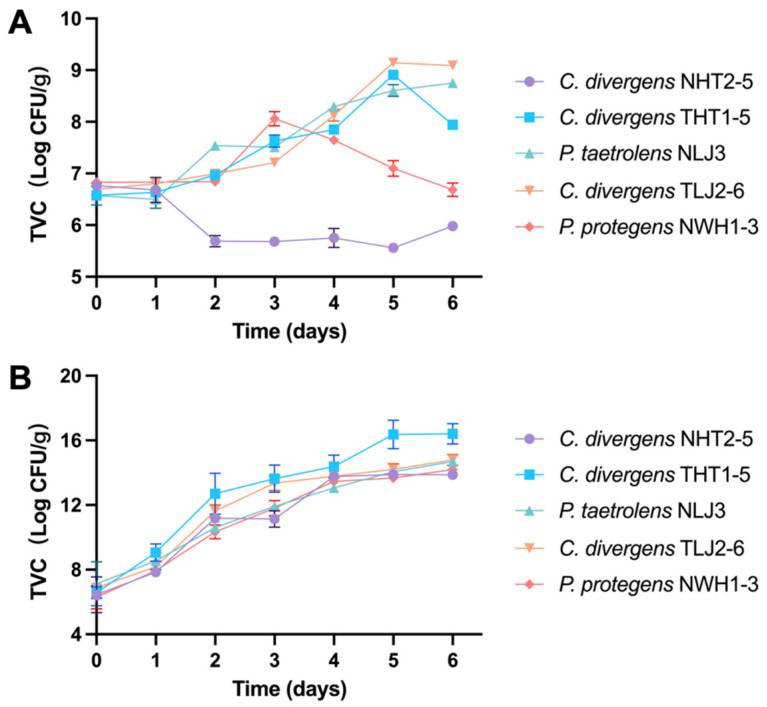
Evolution of total viable bacteria counts (TVCs) (**A**) and total volatile basic nitrogen (TVB-N) content (**B**) in different cuts of black pork (ham, loin, and belly) inoculated with isolated strains during storage at 4 °C. The strains *Carnobacterium divergens* NHT2-5 and THT1-5 were inoculated separately with sterile ham, the strains *Pseudomonas taetrolens* NLJ3 and *C. divergens* TLJ2-6 were dividually inoculated with sterile loin, and the strain *P. protegens* NWH1-3 was inoculated with sterile belly.

In Figure 6, the TVC and TVB-N content in pork cuts inoculated with isolated strains during storage at 4 °C were investigated. Regarding TVC (Figure 6A), samples inoculated with different isolated strains exhibited that TVC levels exceeded 10^6^ (Log CFU/g) on the initial day. The TVC of ham samples inoculated with *C. divergens* NHT2-5 showed a decreasing trend, while the TVC of loin samples inoculated with *P. taetrolens* NLJ3 displayed an increasing trend. Conversely, the TVC of the other three samples initially increased before decreasing over the storage period. Notably, the TVC of loin samples inoculated with *C. divergens* TLJ2-6 reached a peak at 9.14 ± 0.06 Log CFU/g on day 5. As the storage days progressed, there was a continuous increase in TVB-N content, with ham samples inoculated with *C. divergens* THT1-5 exceeding 15 mg/100 g on day 6 (Figure 6B). These results suggested that *C. divergens* THT1-5 exhibited cold adaptation and a higher spoilage potential in Enshi black pork.

## 4. Conclusions

In this study, Enshi black pork cuts, including ham, loin, and belly, were found to have reached spoilage after four days of storage at 4 °C. The bacterial community diversity and composition in spoiled ham, loin, and belly differed significantly (*p* < 0.05), with 20 genera identified as potential bacterial biomarkers. A total of 50 strains representing 14 genera were isolated from the spoiled samples of ham, loin, and belly. Among these, *C. divergens* THT1-5, isolated from spoiled ham samples, exhibited cold adaptation and a higher spoilage potential in Enshi black pork. These findings contribute to the identification of key factors responsible for spoilage and enhance the understanding of the spoilage mechanism in Enshi black pork. Such insights will facilitate the development of strategies to inhibit bacterial growth during the preservation process of Enshi black pork.

## Figures and Tables

**Table 1:** Identification of isolated strains in spoiled black pork.

Samples	Numbers of Isolated Strains	Strain Identification		
		Genera	Species	Strains
Ham	20	*Pseudomonas*	*P. versuta*	THT2-3
		*Acinetobacter*	*A. wanghuae*	THT2-1
		*Carnobacterium*	*C. divergens*	NHT2-5, THT1-5, NHT1-1
			*C. maltaromaticum*	TWH1-2
		*Serratia*	*S. liquefaciens*	NHT1-5, NHT4
		*Bacillus*	*B. licheniformis*	THT3-5
		*Buttiauxella*	*B. gaviniae*	NHT3-2-2, PHT3
		*Hafnia*	*H. alvei*	PHT5
			*H. paralvei*	NHT3-4, THT1-4
		*Klebsiella*	*K. quasivariicola*	NHT2-4
		*Kocuria*	*K. rhizophila*	NHT2-1, NHT1-4
		*Micrococcus*	*M. luteus*	THT2-2
		*Staphylococcus*	*S. saprophyticus*	THT3-1, NHT3-5
Loin	14	*Pseudomonas*	*P. taetrolens*	NLJ3
		*Carnobacterium*	*C. divergens*	TLJ2-6, NLJ1, PLJ1, PLJ2
			*C. maltaromaticum*	TLJ3-4
		*Serratia*	*S. liquefaciens*	NLJ7, TLJ1-5, TLJ2-4, TLJ1-3
		*Hafnia*	*H. alvei*	NLJ4, TLJ1-2
		*Kocuria*	*K. rhizophila*	TLJ1-1, NLJ2
Belly	16	*Pseudomonas*	*P. protegens*	NWH1-3
		*Acinetobacter*	*A. wanghuae*	PWH1-1
		*Carnobacterium*	*C. divergens*	NWH3-3, NWH1-5
		*Serratia*	*S. liquefaciens*	PWH1-3, PWH2-1, TWH2-4
			*S. quinivorans*	NWH3-4
		*Aeromonas*	*A. caviae*	NWH2-2
			*A. media*	NWH1-1
			*A. salmonicida*	NWH1-4, NWH3-5, TWH2-3
		*Kurthia*	*K. zopfii*	NWH2-5
		*Macrococcus*	*M. caseolyticus*	NWH1-2, TWH2-1

## Data Availability

The original contributions presented in the study are included in the article/Supplementary Material, further inquiries can be directed to the corresponding author.

## Supplementary Materials

The following supporting information can be downloaded at https://www.mdpi.com/article/10.3390/foods13132081/s1: Figure S1: Heatmap of top genera based on relative abundance in spoiled black pork; Figure S2: Flower plot based on OUTs in spoiled black pork.
