# Supplementary material for: Behavior of Spoilage Bacterial Communities in Different Cuts of Enshi Black Pork under Refrigerated Storage (4 °C)
Source: Foods. 2024 Jul 1;13(13):2081. doi: 10.3390/foods13132081 (PMC11241765; doi:10.3390/foods13132081)
Supplement: Supplementary file 1 [file foods-13-02081-s001.zip › foods-3045156-supplementary.pdf]

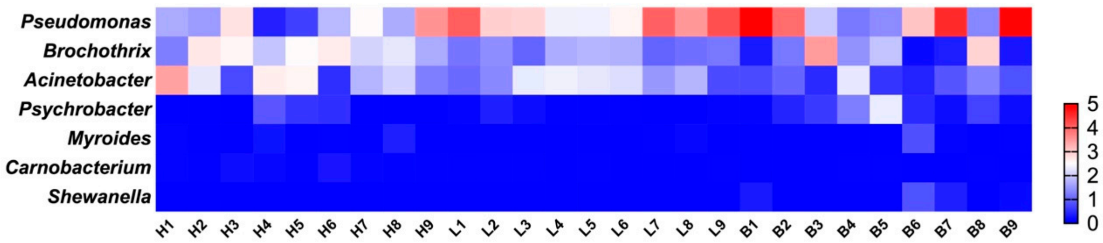

Figure S1. Heatmap of top genera based on relative abundance in spoiled black pork. H, ham; L, loin; B, belly. 9 replicates were represented by the numbers 1-9.

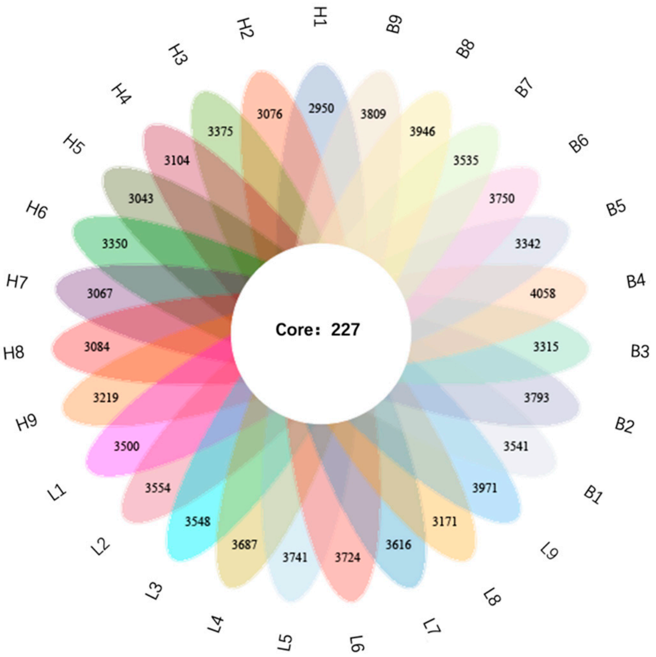

Figure S2. Flower plot based on OUTs in spoiled black pork. H, ham; L, loin; B, belly. 9 replicates were represented by the numbers 1-9.
